# 2,6-Bis(3-fluoro­phen­yl)-3-isopropyl­piperidin-4-one

**DOI:** 10.1107/S1600536810024414

**Published:** 2010-06-30

**Authors:** R. Ramachandran, M. Rani, S. Kabilan, Yeon Tae Jeong

**Affiliations:** a Department of Image Science and Engineering, Pukyong National University, Busan 608 739, Republic of Korea; b Department of Chemistry, Annamalai University, Annamalai Nagar 608 002, Tamil Nadu, India

## Abstract

In the title compound, C_20_H_21_F_2_NO, the piperidine ring in each of the two independent mol­ecules in the asymmetric unit adopts a normal chair conformation with an equatorial orientation of the 3-fluoro­phenyl groups. The dihedral angles between the two 3-fluoro­phenyl rings are 49.89 (7) and 50.35 (7)° in the two mol­ecules.

## Related literature

For background to piperidine-4-ones and related structures, see: Noller & Baliah (1948 ▶); Gayathri *et al.* (2008 ▶); Ramachandran *et al.* (2007 ▶); Pandiarajan *et al.* (1986 ▶). For ring conformational analysis, see: Cremer & Pople (1975 ▶); Nardelli (1983 ▶).
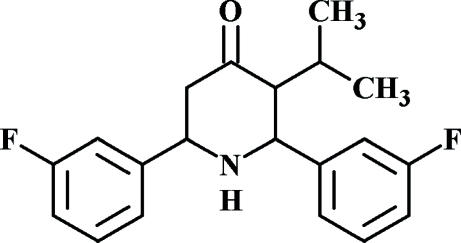


## Experimental

### 

#### Crystal data

C_20_H_21_F_2_NO
*M* *_r_* = 329.38
Monoclinic, 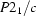

*a* = 8.8217 (3) Å
*b* = 12.7612 (4) Å
*c* = 30.8613 (9) Å
β = 91.892 (2)°
*V* = 3472.33 (19) Å^3^
*Z* = 8
Mo *K*α radiation
μ = 0.09 mm^−1^
*T* = 292 K
0.30 × 0.25 × 0.20 mm

#### Data collection

Bruker Kappa APEXII CCD diffractometer
Absorption correction: multi-scan (*SADABS*; Bruker, 1999 ▶) *T*_min_ = 0.893, *T*_max_ = 0.982
39632 measured reflections
8517 independent reflections
4760 reflections with *I* > 2σ(*I*)
*R*_int_ = 0.051

#### Refinement

*R*[*F* ^2^ > 2σ(*F* ^2^)] = 0.057
*wR*(*F* ^2^) = 0.178
*S* = 1.01
8517 reflections
445 parameters
H atoms treated by a mixture of independent and constrained refinement
Δρ_max_ = 0.36 e Å^−3^
Δρ_min_ = −0.26 e Å^−3^

### 

Data collection: *APEX2* (Bruker, 2004 ▶); cell refinement: *SAINT-Plus* (Bruker, 2004 ▶); data reduction: *SAINT-Plus*; program(s) used to solve structure: *SIR92* (Altomare *et al.*, 1993 ▶); program(s) used to refine structure: *SHELXL97* (Sheldrick, 2008 ▶); molecular graphics: *ORTEP-3* (Farrugia, 1997 ▶); software used to prepare material for publication: *SHELXL97*.

## Supplementary Material

Crystal structure: contains datablocks global, I. DOI: 10.1107/S1600536810024414/rk2203sup1.cif

Structure factors: contains datablocks I. DOI: 10.1107/S1600536810024414/rk2203Isup2.hkl

Additional supplementary materials:  crystallographic information; 3D view; checkCIF report

## Figures and Tables

**Fig. 1.**
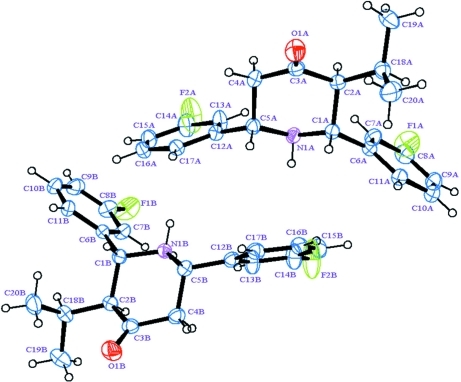
The molecular structure of title compound with the atom numbering scheme. Displacement ellipsoids are shown at 30% probability level. H atoms are presented as a small spheres of arbitrary radius.

## supplementary crystallographic information

### Comment

Heteroatom containing six-membered cyclic compounds possess interesting stereochemistry such as conformation of the ring and orientation of the substituents. Several piperidin-4-ones were reported and analyzed their ring conformations by Noller & Baliah, 1948; Pandiarajan *et al.*, 1986. The present investigation was undertaken to establish the structure, conformation of the heterocyclic ring and orientation of the 3-fluorophenyl groups by X-ray diffraction analysis.

In the molecular structure of title compound, six-membered heterocyclic ring (Fig.1) adopts normal chair conformation with the puckering parameters (Cremer & Pople, 1975) and the smallest displacement asymmetry parameters (Nardelli, 1983) being q_1_ and q_2_ are 0.082 (2)Å and -0.577 (2)Å, respectively. The total puckering amplitude, Q_T_=0.5823 (19)Å; θ=171.9 (2)°. The dihedral angle between the 3-fluorophenyl rings is 49.89 (7)° and 50.35 (7)° of molecule A and B respectively.

### Experimental

The title compound was prepared by the condensation of 4-methylpentan-2-one, 3-fluorobenzaldehyde and ammonium acetate in 1:2:1 molar ratio as reported by Gayathri *et al.*, 2008; Ramachandran *et al.*, 2007. Diffraction quality crystals were obtained by recrystalization of the crude sample from ethanol.

### Refinement

The hydrogen atoms were positioned and refined using a riding model, with aromatic C—H = 0.93Å, methine C—H = 0.98Å, methylene C—H = 0.97Å and methyl C—H = 0.96Å. The displacement parameters were set for phenyl, methylene and aliphatic H atoms at *U*_iso_(H) = 1.2*U*_eq_(C) and 1.5*U*_eq_(methyl C).

### Crystal data

**Table:**

C_20_H_21_F_2_NO	*F*(000) = 1392
*M**_r_* = 329.38	*D*_x_ = 1.260 Mg m^−^^3^
Monoclinic, *P*2_1_/*c*	Mo *K*α radiation, λ = 0.71073 Å
Hall symbol: -P 2ybc	Cell parameters from 6010 reflections
*a* = 8.8217 (3) Å	θ = 2.4–28.1°
*b* = 12.7612 (4) Å	µ = 0.09 mm^−^^1^
*c* = 30.8613 (9) Å	*T* = 292 K
β = 91.892 (2)°	Block, colourless
*V* = 3472.33 (19) Å^3^	0.30 × 0.25 × 0.20 mm
*Z* = 8	

### Data collection

**Table:**

Bruker Kappa APEXII CCD diffractometer	8517 independent reflections
Radiation source: fine-focus sealed tube	4760 reflections with *I* > 2σ(*I*)
graphite	*R*_int_ = 0.051
ω and φ scans	θ_max_ = 28.2°, θ_min_ = 1.3°
Absorption correction: multi-scan (*SADABS*; Bruker, 1999)	*h* = −11→11
*T*_min_ = 0.893, *T*_max_ = 0.982	*k* = −16→14
39632 measured reflections	*l* = −40→41

### Refinement

**Table:**

Refinement on *F*^2^	Primary atom site location: structure-invariant direct methods
Least-squares matrix: full	Secondary atom site location: difference Fourier map
*R*[*F*^2^ > 2σ(*F*^2^)] = 0.057	Hydrogen site location: inferred from neighbouring sites
*wR*(*F*^2^) = 0.178	H atoms treated by a mixture of independent and constrained refinement
*S* = 1.01	*w* = 1/[σ^2^(*F*_o_^2^) + (0.0848*P*)^2^ + 0.3074*P*] where *P* = (*F*_o_^2^ + 2*F*_c_^2^)/3
8517 reflections	(Δ/σ)_max_ = 0.001
445 parameters	Δρ_max_ = 0.36 e Å^−^^3^
0 restraints	Δρ_min_ = −0.26 e Å^−^^3^

### Special details

**Table:**

Geometry. All s.u.'s (except the s.u. in the dihedral angle between two l.s. planes) are estimated using the full covariance matrix. The cell s.u.'s are taken into account individually in the estimation of s.u.'s in distances, angles and torsion angles; correlations between s.u.'s in cell parameters are only used when they are defined by crystal symmetry. An approximate (isotropic) treatment of cell s.u.'s is used for estimating s.u.'s involving l.s. planes.
Refinement. Refinement of *F*^2^ against ALL reflections. The weighted *R*-factor w*R* and goodness of fit *S* are based on *F*^2^, conventional *R*-factors *R* are based on *F*, with *F* set to zero for negative *F*^2^. The threshold expression of *F*^2^ > σ(*F*^2^) is used only for calculating *R*-factors(gt) *etc*. and is not relevant to the choice of reflections for refinement. *R*-factors based on *F*^2^ are statistically about twice as large as those based on *F*, and *R*-factors based on ALL data will be even larger.

### Fractional atomic coordinates and isotropic or equivalent isotropic displacement parameters (Å^2^)

**Table:**

	*x*	*y*	*z*	*U*_iso_*/*U*_eq_	
C1A	0.5616 (2)	0.27192 (15)	0.87176 (6)	0.0366 (4)	
H1A	0.6150	0.3388	0.8688	0.044*	
C1B	0.9557 (2)	0.30455 (15)	1.11617 (6)	0.0371 (4)	
H1B	0.9030	0.3722	1.1149	0.045*	
C2A	0.3926 (2)	0.28694 (15)	0.85761 (6)	0.0364 (4)	
H2A	0.3414	0.2210	0.8643	0.044*	
C2B	1.1232 (2)	0.32290 (15)	1.13062 (6)	0.0388 (5)	
H2B	1.1740	0.2550	1.1281	0.047*	
C3A	0.3289 (2)	0.36851 (16)	0.88737 (6)	0.0412 (5)	
C3B	1.1915 (2)	0.39359 (16)	1.09703 (7)	0.0427 (5)	
C4A	0.3518 (2)	0.34547 (17)	0.93482 (6)	0.0451 (5)	
H4A1	0.2892	0.2862	0.9424	0.054*	
H4A2	0.3195	0.4055	0.9515	0.054*	
C4B	1.1707 (2)	0.35693 (17)	1.05111 (7)	0.0475 (5)	
H4B1	1.2337	0.2958	1.0468	0.057*	
H4B2	1.2041	0.4115	1.0318	0.057*	
C5A	0.5175 (2)	0.32094 (15)	0.94670 (6)	0.0395 (5)	
H5A	0.5788	0.3842	0.9430	0.047*	
C5B	1.0058 (2)	0.32934 (16)	1.03953 (6)	0.0403 (5)	
H5B	0.9445	0.3934	1.0395	0.048*	
C6A	0.6404 (2)	0.18931 (15)	0.84562 (6)	0.0377 (5)	
C6B	0.8732 (2)	0.23227 (15)	1.14601 (6)	0.0377 (5)	
C7A	0.6125 (2)	0.08430 (17)	0.85276 (7)	0.0485 (5)	
H7A	0.5421	0.0633	0.8728	0.058*	
C7B	0.8963 (2)	0.12508 (17)	1.14395 (7)	0.0482 (5)	
H7B	0.9655	0.0970	1.1250	0.058*	
C8A	0.6905 (3)	0.01198 (18)	0.82973 (8)	0.0595 (6)	
C8B	0.8156 (3)	0.06144 (18)	1.17018 (8)	0.0571 (6)	
C9A	0.7939 (3)	0.0373 (2)	0.79971 (8)	0.0613 (7)	
H9A	0.8445	−0.0145	0.7847	0.074*	
C9B	0.7152 (3)	0.0976 (2)	1.19913 (7)	0.0612 (7)	
H9B	0.6634	0.0519	1.2168	0.073*	
C10A	0.8211 (3)	0.1411 (2)	0.79231 (7)	0.0575 (6)	
H10A	0.8909	0.1610	0.7719	0.069*	
C10B	0.6930 (3)	0.2037 (2)	1.20142 (7)	0.0571 (6)	
H10B	0.6248	0.2308	1.2209	0.069*	
C11A	0.7448 (2)	0.21651 (18)	0.81522 (6)	0.0465 (5)	
H11A	0.7641	0.2870	0.8101	0.056*	
C11B	0.7709 (2)	0.27066 (18)	1.17510 (6)	0.0466 (5)	
H11B	0.7546	0.3425	1.1769	0.056*	
C12A	0.5327 (2)	0.28483 (16)	0.99315 (6)	0.0408 (5)	
C12B	0.9932 (2)	0.27947 (16)	0.99536 (6)	0.0425 (5)	
C13A	0.5026 (3)	0.18218 (18)	1.00394 (7)	0.0600 (6)	
H13A	0.4792	0.1330	0.9825	0.072*	
C13B	1.0278 (3)	0.17523 (18)	0.98992 (7)	0.0651 (7)	
H13B	1.0536	0.1332	1.0136	0.078*	
C14A	0.5078 (3)	0.15399 (19)	1.04681 (8)	0.0669 (7)	
C14B	1.0231 (4)	0.13474 (19)	0.94869 (8)	0.0729 (8)	
C15A	0.5410 (3)	0.2215 (2)	1.07965 (7)	0.0580 (6)	
H15A	0.5438	0.1993	1.1084	0.070*	
C15B	0.9867 (3)	0.1915 (2)	0.91260 (7)	0.0621 (7)	
H15B	0.9845	0.1610	0.8852	0.074*	
C16A	0.5700 (2)	0.32323 (19)	1.06900 (7)	0.0532 (6)	
H16A	0.5914	0.3719	1.0908	0.064*	
C16B	0.9538 (3)	0.2944 (2)	0.91814 (7)	0.0555 (6)	
H16B	0.9301	0.3359	0.8941	0.067*	
C17A	0.5677 (2)	0.35430 (17)	1.02624 (6)	0.0457 (5)	
H17A	0.5901	0.4235	1.0195	0.055*	
C17B	0.9551 (2)	0.33769 (18)	0.95890 (7)	0.0488 (5)	
H17B	0.9298	0.4080	0.9620	0.059*	
C18A	0.3643 (2)	0.30560 (16)	0.80898 (6)	0.0449 (5)	
H18A	0.4135	0.2477	0.7940	0.054*	
C18B	1.1476 (2)	0.35704 (17)	1.17792 (6)	0.0479 (5)	
H18B	1.0948	0.3055	1.1955	0.057*	
C19A	0.1956 (3)	0.29873 (19)	0.79675 (8)	0.0609 (6)	
H19A	0.1428	0.3554	0.8101	0.091*	
H19B	0.1560	0.2332	0.8066	0.091*	
H19C	0.1818	0.3033	0.7658	0.091*	
C19B	1.3150 (3)	0.3509 (2)	1.19177 (8)	0.0688 (7)	
H19D	1.3512	0.2808	1.1875	0.103*	
H19E	1.3270	0.3692	1.2219	0.103*	
H19F	1.3720	0.3987	1.1747	0.103*	
C20A	0.4336 (3)	0.4060 (2)	0.79204 (7)	0.0645 (7)	
H20A	0.4177	0.4094	0.7612	0.097*	
H20B	0.5405	0.4066	0.7990	0.097*	
H20C	0.3866	0.4652	0.8052	0.097*	
C20B	1.0808 (3)	0.46335 (18)	1.18874 (8)	0.0662 (7)	
H20D	1.0878	0.4742	1.2195	0.099*	
H20E	0.9764	0.4656	1.1791	0.099*	
H20F	1.1362	0.5173	1.1745	0.099*	
N1A	0.5684 (2)	0.23981 (13)	0.91734 (5)	0.0394 (4)	
N1B	0.9526 (2)	0.25835 (13)	1.07272 (5)	0.0403 (4)	
O1A	0.26770 (19)	0.44834 (12)	0.87504 (5)	0.0625 (5)	
O1B	1.2548 (2)	0.47534 (12)	1.10547 (5)	0.0654 (5)	
F1A	0.6646 (2)	−0.09068 (12)	0.83741 (6)	0.1012 (6)	
F1B	0.8356 (2)	−0.04357 (12)	1.16716 (6)	0.0985 (6)	
F2A	0.4787 (3)	0.05316 (13)	1.05680 (5)	0.1244 (8)	
F2B	1.0578 (3)	0.03231 (13)	0.94412 (6)	0.1389 (10)	
H1D	0.856 (3)	0.2386 (16)	1.0657 (7)	0.052 (6)*	
H1C	0.669 (3)	0.2223 (17)	0.9257 (7)	0.058 (7)*	

### Atomic displacement parameters (Å^2^)

**Table:**

	*U*^11^	*U*^22^	*U*^33^	*U*^12^	*U*^13^	*U*^23^
C1A	0.0388 (11)	0.0361 (11)	0.0351 (10)	−0.0011 (8)	0.0032 (8)	−0.0007 (8)
C1B	0.0381 (11)	0.0332 (11)	0.0402 (10)	0.0023 (8)	0.0014 (8)	−0.0025 (8)
C2A	0.0365 (11)	0.0328 (10)	0.0399 (10)	−0.0015 (8)	0.0015 (8)	0.0006 (8)
C2B	0.0387 (11)	0.0322 (11)	0.0451 (11)	0.0026 (8)	−0.0022 (9)	−0.0017 (8)
C3A	0.0369 (11)	0.0395 (12)	0.0471 (11)	0.0010 (9)	0.0012 (9)	−0.0022 (9)
C3B	0.0375 (11)	0.0370 (12)	0.0536 (12)	−0.0008 (9)	0.0014 (9)	−0.0025 (9)
C4A	0.0465 (13)	0.0485 (13)	0.0409 (11)	0.0042 (10)	0.0078 (9)	−0.0069 (9)
C4B	0.0451 (13)	0.0474 (13)	0.0505 (12)	−0.0076 (10)	0.0107 (10)	0.0012 (10)
C5A	0.0433 (12)	0.0376 (11)	0.0377 (10)	−0.0043 (9)	0.0039 (8)	−0.0043 (8)
C5B	0.0419 (12)	0.0388 (11)	0.0404 (11)	0.0031 (9)	0.0055 (9)	0.0008 (8)
C6A	0.0354 (11)	0.0430 (12)	0.0344 (10)	0.0009 (9)	−0.0014 (8)	−0.0012 (8)
C6B	0.0359 (11)	0.0428 (12)	0.0343 (10)	−0.0007 (9)	−0.0015 (8)	−0.0021 (8)
C7A	0.0498 (13)	0.0427 (13)	0.0534 (13)	0.0014 (10)	0.0071 (10)	−0.0024 (10)
C7B	0.0456 (13)	0.0454 (13)	0.0544 (13)	0.0015 (10)	0.0131 (10)	0.0013 (10)
C8A	0.0668 (17)	0.0393 (14)	0.0718 (16)	0.0071 (12)	−0.0052 (13)	−0.0091 (11)
C8B	0.0586 (15)	0.0436 (14)	0.0693 (15)	−0.0014 (11)	0.0083 (12)	0.0116 (11)
C9A	0.0523 (15)	0.0726 (18)	0.0590 (15)	0.0171 (13)	0.0010 (12)	−0.0252 (13)
C9B	0.0526 (15)	0.0786 (19)	0.0529 (14)	−0.0085 (13)	0.0094 (11)	0.0169 (12)
C10A	0.0488 (14)	0.0801 (19)	0.0438 (12)	0.0081 (12)	0.0051 (10)	−0.0104 (12)
C10B	0.0472 (14)	0.0838 (19)	0.0409 (12)	0.0005 (12)	0.0106 (10)	−0.0040 (12)
C11A	0.0433 (12)	0.0547 (14)	0.0415 (11)	0.0016 (10)	0.0022 (9)	0.0001 (10)
C11B	0.0457 (13)	0.0539 (14)	0.0404 (11)	0.0043 (10)	0.0037 (9)	−0.0081 (9)
C12A	0.0407 (11)	0.0427 (12)	0.0390 (10)	0.0001 (9)	0.0030 (8)	−0.0031 (9)
C12B	0.0414 (12)	0.0428 (12)	0.0437 (11)	−0.0042 (9)	0.0060 (9)	0.0010 (9)
C13A	0.0956 (19)	0.0450 (14)	0.0399 (12)	−0.0079 (13)	0.0074 (12)	−0.0055 (10)
C13B	0.106 (2)	0.0470 (14)	0.0424 (13)	0.0049 (14)	0.0072 (13)	0.0029 (10)
C14A	0.101 (2)	0.0508 (16)	0.0496 (14)	−0.0053 (14)	0.0073 (13)	0.0063 (11)
C14B	0.122 (2)	0.0415 (15)	0.0561 (15)	−0.0038 (14)	0.0135 (15)	−0.0087 (11)
C15A	0.0604 (15)	0.0767 (18)	0.0368 (11)	0.0055 (13)	−0.0013 (10)	0.0026 (11)
C15B	0.0673 (17)	0.0756 (19)	0.0434 (13)	−0.0119 (14)	0.0030 (11)	−0.0074 (12)
C16A	0.0474 (13)	0.0684 (17)	0.0433 (12)	0.0056 (11)	−0.0069 (10)	−0.0156 (11)
C16B	0.0502 (14)	0.0731 (17)	0.0429 (12)	−0.0033 (12)	−0.0043 (10)	0.0073 (11)
C17A	0.0414 (12)	0.0494 (13)	0.0461 (12)	0.0012 (10)	−0.0017 (9)	−0.0087 (9)
C17B	0.0452 (13)	0.0516 (14)	0.0495 (13)	−0.0010 (10)	−0.0010 (10)	0.0047 (10)
C18A	0.0494 (13)	0.0453 (12)	0.0396 (11)	0.0094 (10)	−0.0031 (9)	−0.0012 (9)
C18B	0.0535 (14)	0.0446 (13)	0.0449 (12)	−0.0043 (10)	−0.0073 (10)	−0.0020 (9)
C19A	0.0599 (16)	0.0618 (16)	0.0596 (14)	0.0046 (12)	−0.0197 (12)	−0.0018 (12)
C19B	0.0661 (17)	0.0626 (17)	0.0757 (17)	−0.0040 (13)	−0.0266 (13)	−0.0016 (13)
C20A	0.0616 (16)	0.0756 (18)	0.0565 (14)	0.0027 (13)	0.0059 (12)	0.0235 (12)
C20B	0.0739 (18)	0.0630 (17)	0.0613 (15)	0.0052 (13)	−0.0056 (13)	−0.0226 (12)
N1A	0.0406 (10)	0.0427 (10)	0.0349 (9)	0.0067 (8)	0.0027 (7)	−0.0009 (7)
N1B	0.0400 (10)	0.0431 (10)	0.0379 (9)	−0.0078 (8)	0.0035 (7)	−0.0030 (7)
O1A	0.0752 (12)	0.0512 (10)	0.0609 (10)	0.0247 (9)	−0.0019 (8)	−0.0015 (8)
O1B	0.0754 (12)	0.0515 (10)	0.0695 (11)	−0.0248 (9)	0.0036 (9)	−0.0024 (8)
F1A	0.1198 (15)	0.0471 (10)	0.1379 (15)	0.0088 (9)	0.0230 (12)	−0.0122 (9)
F1B	0.1071 (14)	0.0511 (10)	0.1397 (15)	−0.0017 (9)	0.0408 (11)	0.0226 (9)
F2A	0.243 (3)	0.0645 (11)	0.0667 (11)	−0.0309 (13)	0.0118 (13)	0.0188 (8)
F2B	0.288 (3)	0.0562 (11)	0.0733 (12)	0.0189 (14)	0.0228 (14)	−0.0154 (8)

### Geometric parameters (Å, °)

**Table:**

C1A—N1A	1.465 (2)	C10A—H10A	0.9300
C1A—C6A	1.511 (3)	C10B—C11B	1.378 (3)
C1A—C2A	1.551 (3)	C10B—H10B	0.9300
C1A—H1A	0.9800	C11A—H11A	0.9300
C1B—N1B	1.464 (2)	C11B—H11B	0.9300
C1B—C6B	1.507 (3)	C12A—C17A	1.379 (3)
C1B—C2B	1.547 (3)	C12A—C13A	1.380 (3)
C1B—H1B	0.9800	C12B—C13B	1.376 (3)
C2A—C3A	1.509 (3)	C12B—C17B	1.381 (3)
C2A—C18A	1.532 (3)	C13A—C14A	1.370 (3)
C2A—H2A	0.9800	C13A—H13A	0.9300
C2B—C3B	1.514 (3)	C13B—C14B	1.373 (3)
C2B—C18B	1.532 (3)	C13B—H13B	0.9300
C2B—H2B	0.9800	C14A—F2A	1.350 (3)
C3A—O1A	1.208 (2)	C14A—C15A	1.354 (3)
C3A—C4A	1.501 (3)	C14B—F2B	1.351 (3)
C3B—O1B	1.208 (2)	C14B—C15B	1.358 (3)
C3B—C4B	1.498 (3)	C15A—C16A	1.366 (3)
C4A—C5A	1.528 (3)	C15A—H15A	0.9300
C4A—H4A1	0.9700	C15B—C16B	1.357 (3)
C4A—H4A2	0.9700	C15B—H15B	0.9300
C4B—C5B	1.528 (3)	C16A—C17A	1.377 (3)
C4B—H4B1	0.9700	C16A—H16A	0.9300
C4B—H4B2	0.9700	C16B—C17B	1.374 (3)
C5A—N1A	1.456 (2)	C16B—H16B	0.9300
C5A—C12A	1.508 (3)	C17A—H17A	0.9300
C5A—H5A	0.9800	C17B—H17B	0.9300
C5B—N1B	1.456 (2)	C18A—C20A	1.520 (3)
C5B—C12B	1.505 (3)	C18A—C19A	1.526 (3)
C5B—H5B	0.9800	C18A—H18A	0.9800
C6A—C11A	1.380 (3)	C18B—C20B	1.520 (3)
C6A—C7A	1.382 (3)	C18B—C19B	1.526 (3)
C6B—C11B	1.383 (3)	C18B—H18B	0.9800
C6B—C7B	1.385 (3)	C19A—H19A	0.9600
C7A—C8A	1.365 (3)	C19A—H19B	0.9600
C7A—H7A	0.9300	C19A—H19C	0.9600
C7B—C8B	1.364 (3)	C19B—H19D	0.9600
C7B—H7B	0.9300	C19B—H19E	0.9600
C8A—F1A	1.352 (3)	C19B—H19F	0.9600
C8A—C9A	1.360 (4)	C20A—H20A	0.9600
C8B—F1B	1.355 (3)	C20A—H20B	0.9600
C8B—C9B	1.359 (3)	C20A—H20C	0.9600
C9A—C10A	1.368 (3)	C20B—H20D	0.9600
C9A—H9A	0.9300	C20B—H20E	0.9600
C9B—C10B	1.370 (3)	C20B—H20F	0.9600
C9B—H9B	0.9300	N1A—H1C	0.94 (2)
C10A—C11A	1.382 (3)	N1B—H1D	0.90 (2)
			
N1A—C1A—C6A	108.22 (15)	C6A—C11A—C10A	121.3 (2)
N1A—C1A—C2A	108.26 (15)	C6A—C11A—H11A	119.3
C6A—C1A—C2A	113.02 (15)	C10A—C11A—H11A	119.3
N1A—C1A—H1A	109.1	C10B—C11B—C6B	120.8 (2)
C6A—C1A—H1A	109.1	C10B—C11B—H11B	119.6
C2A—C1A—H1A	109.1	C6B—C11B—H11B	119.6
N1B—C1B—C6B	108.57 (15)	C17A—C12A—C13A	118.14 (19)
N1B—C1B—C2B	108.26 (15)	C17A—C12A—C5A	121.22 (19)
C6B—C1B—C2B	113.08 (15)	C13A—C12A—C5A	120.51 (17)
N1B—C1B—H1B	109.0	C13B—C12B—C17B	118.02 (19)
C6B—C1B—H1B	109.0	C13B—C12B—C5B	120.61 (18)
C2B—C1B—H1B	109.0	C17B—C12B—C5B	121.27 (19)
C3A—C2A—C18A	115.91 (16)	C14A—C13A—C12A	118.8 (2)
C3A—C2A—C1A	106.73 (15)	C14A—C13A—H13A	120.6
C18A—C2A—C1A	114.78 (16)	C12A—C13A—H13A	120.6
C3A—C2A—H2A	106.2	C14B—C13B—C12B	118.5 (2)
C18A—C2A—H2A	106.2	C14B—C13B—H13B	120.8
C1A—C2A—H2A	106.2	C12B—C13B—H13B	120.8
C3B—C2B—C18B	115.87 (17)	F2A—C14A—C15A	118.2 (2)
C3B—C2B—C1B	106.91 (15)	F2A—C14A—C13A	118.0 (2)
C18B—C2B—C1B	114.87 (16)	C15A—C14A—C13A	123.7 (2)
C3B—C2B—H2B	106.1	F2B—C14B—C15B	118.6 (2)
C18B—C2B—H2B	106.1	F2B—C14B—C13B	117.5 (2)
C1B—C2B—H2B	106.1	C15B—C14B—C13B	124.0 (2)
O1A—C3A—C4A	121.10 (19)	C14A—C15A—C16A	117.5 (2)
O1A—C3A—C2A	124.16 (19)	C14A—C15A—H15A	121.2
C4A—C3A—C2A	114.71 (17)	C16A—C15A—H15A	121.2
O1B—C3B—C4B	121.01 (19)	C16B—C15B—C14B	117.3 (2)
O1B—C3B—C2B	123.97 (19)	C16B—C15B—H15B	121.4
C4B—C3B—C2B	114.99 (17)	C14B—C15B—H15B	121.4
C3A—C4A—C5A	111.77 (16)	C15A—C16A—C17A	120.5 (2)
C3A—C4A—H4A1	109.3	C15A—C16A—H16A	119.8
C5A—C4A—H4A1	109.3	C17A—C16A—H16A	119.8
C3A—C4A—H4A2	109.3	C15B—C16B—C17B	120.6 (2)
C5A—C4A—H4A2	109.3	C15B—C16B—H16B	119.7
H4A1—C4A—H4A2	107.9	C17B—C16B—H16B	119.7
C3B—C4B—C5B	112.31 (17)	C16A—C17A—C12A	121.3 (2)
C3B—C4B—H4B1	109.1	C16A—C17A—H17A	119.3
C5B—C4B—H4B1	109.1	C12A—C17A—H17A	119.3
C3B—C4B—H4B2	109.1	C16B—C17B—C12B	121.6 (2)
C5B—C4B—H4B2	109.1	C16B—C17B—H17B	119.2
H4B1—C4B—H4B2	107.9	C12B—C17B—H17B	119.2
N1A—C5A—C12A	110.77 (16)	C20A—C18A—C19A	111.35 (17)
N1A—C5A—C4A	107.98 (15)	C20A—C18A—C2A	114.42 (17)
C12A—C5A—C4A	110.20 (16)	C19A—C18A—C2A	111.09 (18)
N1A—C5A—H5A	109.3	C20A—C18A—H18A	106.5
C12A—C5A—H5A	109.3	C19A—C18A—H18A	106.5
C4A—C5A—H5A	109.3	C2A—C18A—H18A	106.5
N1B—C5B—C12B	110.99 (16)	C20B—C18B—C19B	111.27 (18)
N1B—C5B—C4B	107.76 (16)	C20B—C18B—C2B	114.81 (17)
C12B—C5B—C4B	110.50 (16)	C19B—C18B—C2B	110.89 (19)
N1B—C5B—H5B	109.2	C20B—C18B—H18B	106.4
C12B—C5B—H5B	109.2	C19B—C18B—H18B	106.4
C4B—C5B—H5B	109.2	C2B—C18B—H18B	106.4
C11A—C6A—C7A	118.61 (19)	C18A—C19A—H19A	109.5
C11A—C6A—C1A	121.09 (18)	C18A—C19A—H19B	109.5
C7A—C6A—C1A	120.27 (18)	H19A—C19A—H19B	109.5
C11B—C6B—C7B	118.67 (19)	C18A—C19A—H19C	109.5
C11B—C6B—C1B	121.19 (18)	H19A—C19A—H19C	109.5
C7B—C6B—C1B	120.11 (18)	H19B—C19A—H19C	109.5
C8A—C7A—C6A	118.5 (2)	C18B—C19B—H19D	109.5
C8A—C7A—H7A	120.8	C18B—C19B—H19E	109.5
C6A—C7A—H7A	120.8	H19D—C19B—H19E	109.5
C8B—C7B—C6B	118.7 (2)	C18B—C19B—H19F	109.5
C8B—C7B—H7B	120.6	H19D—C19B—H19F	109.5
C6B—C7B—H7B	120.6	H19E—C19B—H19F	109.5
F1A—C8A—C9A	118.0 (2)	C18A—C20A—H20A	109.5
F1A—C8A—C7A	118.2 (2)	C18A—C20A—H20B	109.5
C9A—C8A—C7A	123.7 (2)	H20A—C20A—H20B	109.5
F1B—C8B—C9B	118.1 (2)	C18A—C20A—H20C	109.5
F1B—C8B—C7B	118.4 (2)	H20A—C20A—H20C	109.5
C9B—C8B—C7B	123.5 (2)	H20B—C20A—H20C	109.5
C8A—C9A—C10A	118.0 (2)	C18B—C20B—H20D	109.5
C8A—C9A—H9A	121.0	C18B—C20B—H20E	109.5
C10A—C9A—H9A	121.0	H20D—C20B—H20E	109.5
C8B—C9B—C10B	117.8 (2)	C18B—C20B—H20F	109.5
C8B—C9B—H9B	121.1	H20D—C20B—H20F	109.5
C10B—C9B—H9B	121.1	H20E—C20B—H20F	109.5
C9A—C10A—C11A	119.8 (2)	C5A—N1A—C1A	113.26 (15)
C9A—C10A—H10A	120.1	C5A—N1A—H1C	107.8 (13)
C11A—C10A—H10A	120.1	C1A—N1A—H1C	109.7 (13)
C9B—C10B—C11B	120.5 (2)	C5B—N1B—C1B	113.39 (15)
C9B—C10B—H10B	119.8	C5B—N1B—H1D	109.1 (13)
C11B—C10B—H10B	119.8	C1B—N1B—H1D	108.6 (13)
			
N1A—C1A—C2A—C3A	58.33 (19)	C9A—C10A—C11A—C6A	−0.1 (3)
C6A—C1A—C2A—C3A	178.18 (16)	C9B—C10B—C11B—C6B	−0.2 (3)
N1A—C1A—C2A—C18A	−171.79 (16)	C7B—C6B—C11B—C10B	−0.2 (3)
C6A—C1A—C2A—C18A	−51.9 (2)	C1B—C6B—C11B—C10B	178.21 (18)
N1B—C1B—C2B—C3B	57.92 (19)	N1A—C5A—C12A—C17A	146.61 (19)
C6B—C1B—C2B—C3B	178.26 (15)	C4A—C5A—C12A—C17A	−94.0 (2)
N1B—C1B—C2B—C18B	−172.02 (16)	N1A—C5A—C12A—C13A	−37.5 (3)
C6B—C1B—C2B—C18B	−51.7 (2)	C4A—C5A—C12A—C13A	81.9 (2)
C18A—C2A—C3A—O1A	−5.0 (3)	N1B—C5B—C12B—C13B	−39.5 (3)
C1A—C2A—C3A—O1A	124.3 (2)	C4B—C5B—C12B—C13B	80.0 (3)
C18A—C2A—C3A—C4A	177.13 (17)	N1B—C5B—C12B—C17B	144.1 (2)
C1A—C2A—C3A—C4A	−53.6 (2)	C4B—C5B—C12B—C17B	−96.4 (2)
C18B—C2B—C3B—O1B	−4.0 (3)	C17A—C12A—C13A—C14A	0.4 (3)
C1B—C2B—C3B—O1B	125.5 (2)	C5A—C12A—C13A—C14A	−175.7 (2)
C18B—C2B—C3B—C4B	178.10 (18)	C17B—C12B—C13B—C14B	0.2 (4)
C1B—C2B—C3B—C4B	−52.4 (2)	C5B—C12B—C13B—C14B	−176.3 (2)
O1A—C3A—C4A—C5A	−126.3 (2)	C12A—C13A—C14A—F2A	−179.7 (2)
C2A—C3A—C4A—C5A	51.7 (2)	C12A—C13A—C14A—C15A	0.1 (4)
O1B—C3B—C4B—C5B	−127.6 (2)	C12B—C13B—C14B—F2B	179.8 (3)
C2B—C3B—C4B—C5B	50.3 (2)	C12B—C13B—C14B—C15B	0.2 (5)
C3A—C4A—C5A—N1A	−51.7 (2)	F2A—C14A—C15A—C16A	−179.9 (2)
C3A—C4A—C5A—C12A	−172.77 (17)	C13A—C14A—C15A—C16A	0.3 (4)
C3B—C4B—C5B—N1B	−51.0 (2)	F2B—C14B—C15B—C16B	−179.4 (3)
C3B—C4B—C5B—C12B	−172.41 (17)	C13B—C14B—C15B—C16B	0.2 (4)
N1A—C1A—C6A—C11A	−133.00 (19)	C14A—C15A—C16A—C17A	−1.1 (4)
C2A—C1A—C6A—C11A	107.1 (2)	C14B—C15B—C16B—C17B	−1.1 (4)
N1A—C1A—C6A—C7A	45.0 (2)	C15A—C16A—C17A—C12A	1.6 (3)
C2A—C1A—C6A—C7A	−74.9 (2)	C13A—C12A—C17A—C16A	−1.2 (3)
N1B—C1B—C6B—C11B	−135.60 (19)	C5A—C12A—C17A—C16A	174.83 (19)
C2B—C1B—C6B—C11B	104.2 (2)	C15B—C16B—C17B—C12B	1.6 (3)
N1B—C1B—C6B—C7B	42.8 (2)	C13B—C12B—C17B—C16B	−1.1 (3)
C2B—C1B—C6B—C7B	−77.3 (2)	C5B—C12B—C17B—C16B	175.4 (2)
C11A—C6A—C7A—C8A	0.6 (3)	C3A—C2A—C18A—C20A	61.5 (2)
C1A—C6A—C7A—C8A	−177.38 (19)	C1A—C2A—C18A—C20A	−63.7 (2)
C11B—C6B—C7B—C8B	1.0 (3)	C3A—C2A—C18A—C19A	−65.6 (2)
C1B—C6B—C7B—C8B	−177.45 (19)	C1A—C2A—C18A—C19A	169.19 (17)
C6A—C7A—C8A—F1A	178.86 (19)	C3B—C2B—C18B—C20B	60.8 (3)
C6A—C7A—C8A—C9A	−0.6 (4)	C1B—C2B—C18B—C20B	−64.8 (2)
C6B—C7B—C8B—F1B	178.2 (2)	C3B—C2B—C18B—C19B	−66.4 (2)
C6B—C7B—C8B—C9B	−1.5 (4)	C1B—C2B—C18B—C19B	168.07 (17)
F1A—C8A—C9A—C10A	−179.3 (2)	C12A—C5A—N1A—C1A	−177.83 (16)
C7A—C8A—C9A—C10A	0.1 (4)	C4A—C5A—N1A—C1A	61.4 (2)
F1B—C8B—C9B—C10B	−178.7 (2)	C6A—C1A—N1A—C5A	170.81 (16)
C7B—C8B—C9B—C10B	1.0 (4)	C2A—C1A—N1A—C5A	−66.4 (2)
C8A—C9A—C10A—C11A	0.2 (3)	C12B—C5B—N1B—C1B	−177.36 (16)
C8B—C9B—C10B—C11B	−0.2 (3)	C4B—C5B—N1B—C1B	61.5 (2)
C7A—C6A—C11A—C10A	−0.3 (3)	C6B—C1B—N1B—C5B	170.01 (16)
C1A—C6A—C11A—C10A	177.70 (18)	C2B—C1B—N1B—C5B	−66.9 (2)
